# The Pathology and Treatment of Extra-Uterine Pregnancy

**Published:** 1885-05

**Authors:** 


### The Pathology and Treatment of Extra-Uterine Pregnancy.

At the meeting of the British Medical Association, Sir Lawson Tait read a paper on this subject, of which the following is an abstract:

He does not believe that ovarian pregnancy ever exists. He believes that all extra-uterine pregnancies are internal. In none of my own dissections or operations on the earlier cases was there the slightest doubt, nor could there be, that the site of pregnancy was the Fallopian tube. Nor, in any record of these cases, is there any doubt about it. In fact, no early extra-uterine pregnancy having any other site than the Fallopian tube has ever been seen, or if so, it has escaped my research.

Under normal circumstances impregnation takes place in the uterus rather than in the tubes, is his teaching, founded upon the facts: (1) that spermatozoa have never been found in the Fallopian tubes or abdominal cavity of the human female; (2) the ciliated epithelium of the Fallopian tubes seem to be an arrangement to prevent the passage of spermatozoa into the Fallopian tubes, thus preventing the occurrences of tubal pregnancy.

As soon as impregnation of the ovum occurs in mammals, the ovule adheres to the mucous surface with which it is in contact. To have a tubal pregnancy, therefore, the ovum must be there when impregnated, and the cause, unquestionably, is due to a desquamative salpingitis. "If we imagine a Fallopian tube to be wholly, or in part, deprived of its vibratile ciliæ, it is not difficult to see how an ovum may become impregnated and rest there, becoming attached and developing into a tubal pregnancy."

Salpingitis is a very common disease, and generally results in occlusion of the tube, thus serving to prevent tubal pregnancy. It is not difficult to believe that the cases in which the attack is limited to the mere destruction of the epithelium must be very rare, and, therefore, we have in this the prime factor in the rarity of tubal pregnancies. The great majority of tubal pregnancies occur in women who have either never had normal pregnancies, or who have not been pregnant for a number of years, so that it is certain that in them the reproductive machinery is out of gear."

Extra-uterine pregnancy, clinically, attracts attention in two phases: (1) By the large number of cases of death from rupture of the tube, resulting in hemorrhage which is fatal; (2) By these few cases in which, after rupture, the fœtus either goes to or approaches the full term of gestation.

The reasons for the removal of the small proportion of patients following rupture are cogent. The site of inplantation of the placenta in the tube is a matter of chance. The tube, necessarily, will be weakened at this point of placental attachment, hence the liability of rupture at this point. Three-fourths of the circumference of the tube is covered by the folds of the broad ligament and the remaining one-fourth on the lower side of the tube looks toward the space between these folds. When the placenta is attached so that rupture occurs toward this three-fourths surface of broad ligaments, the fœtus and blood must, necessarily, escape into the peritoneal cavity and death ensue. Should the placenta be attached so that rupture occurs between the folds of the broad ligament, then the space between would serve to hold the fœtus and extruded blood, and prevent fatal hemorrhage because of the confined space.

Mr. Tait says: "It seems to me that when a pregnancy has developed in the tube so far as the twelfth or thirteenth week, the tube can be distended no more, and its walls give way. This rupture, however, may occur as early as the eighth week, though usually it occurs at the tenth or twelfth."

The ovum may perish with the rupture. Then only a small hematocele remains to be absorbed or treated surgically. The ovum may live and develop. "The fœtus often dies about the fifth month, and the cyst suppurates." It then either ruptures into the rectum or cœcum, generally. If the child goes on to maturity, it ordinarily dies after a spurious labor. Operation at the time of rupture has been followed by a successful issue in 80 per cent. of the cases treated by Mr. Tait. If extra-uterine pregnancy advances to nearly full term and the diagnosis is made, operation is demanded. If the child is dead, operation is still necessary, the cardinal features of which are: "(1) To stitch the opening of the sac carefully to the wound in the abdomen so as to close the peritoneal cavity accurately; (2) To leave the placenta entirely alone, and allow it to be thrown off by nature's own processes." The latter operation has been uniformly successful in Mr. Tait's hands.

